# Synthesis of palaeoecological data from the Polish Lowlands suggests heterogeneous patterns of old-growth forest loss after the Migration Period

**DOI:** 10.1038/s41598-022-12241-1

**Published:** 2022-05-20

**Authors:** Sambor Czerwiński, Katarzyna Marcisz, Agnieszka Wacnik, Mariusz Lamentowicz

**Affiliations:** 1 Climate Change Ecology Research Unit, Faculty of Geographical and Geological Sciences, Adam Mickiewicz University, Bogumiła Krygowskiego 10, 61-680 Poznań, Poland; 2 Max Planck Institute for the Science of Human History, Kahlaische Strasse 10, 07745 Jena, Germany; 3 W. Szafer Institute of Botany Polish Academy of Sciences, Lubicz 46, 31-512 Kraków, Poland

**Keywords:** Biogeography, Forest ecology, Palaeoecology

## Abstract

Human impact on Central European forests dates back thousands of years. In this study we reanalyzed 36 published pollen data sets with robust chronologies from Polish Lowlands to determine the patterns of large-scale forest decline after the Migration Period (fourth to sixth century CE). The study revealed substantial heterogeneity in the old-growth forest decline patterns. Using new high-resolution studies, we could better understand the timing of this transition related to increasing economic development. After the Migration Period, forest expansion continued until the seventh to ninth centuries cal. CE, when the dawn of Slavic culture resulted in large-scale forest decline, especially in north-western and north-central Poland. Later, forest decline was recorded mainly in north-eastern Poland and was related to Prussian settlements, including activities associated with the Teutonic Order, as well as with new settlements from the fourteenth century. The composite picture shows a varied spatio-temporal forest loss and transition towards the present-day, human activity dominated landscapes. However, some sites, such as in north-eastern Poland, are characterized by a less abrupt critical transition. The pristine nature of the oak-hornbeam forest had already been destroyed in Early Medieval times (eighth to ninth centuries cal. CE) and the potential for recovery was largely lost. Our study has confirmed previous assumptions that the decline of hornbeam across the Polish Lowlands may be an early indicator of local settlement processes, preceding severe forest loss, and establishment of permanent agriculture.

## Introduction

Human impact has been identified as one of the primary drivers of changes in temperate forest cover in Central Europe during the Holocene^1–8^. In general, the greater the human pressure in the temperate forest zone, the smaller the forest area^5,9,10^. However, in some instances, sudden events associated with climate and socioeconomic transitions, often caused by warfare and/or epidemics, have resulted in restricted impact of human activity on vegetation^11–14^. The Migration Period (MP) was, without a doubt, the last such large-scale event in Central Europe^11^. In addition to political and ethnic transformations in Europe^11,15,16^, it was a period of brief respite for the vegetation, especially in Central Europe^17–20^. Within Poland, this period is assumed to have lasted from the second half of the fourth century until the beginning of the sixth century CE (from this moment, CE should be assumed where the era is not indicated). Still, its range and chronology are inferred from limited archaeological finds^21^. Palynological data revealed that the forest started to grow on fallow and abandoned pastures across the Polish Lowlands during the MP cf.^22–24^. This forest regeneration was characterized by a spread of European hornbeam (*Carpinus betulus*) and common beech (*Fagus sylvatica*, mainly in NW Poland). The regenerated forest was then probably gradually exploited by new Slavic societies from the east, western Ukraine and southern Belarus. They started to colonize the ‘empty’ landscape and thus renewed forest clearances^25,26^. However, until ca. fifteenth century, the north-eastern part of the Polish Lowlands was occupied by diverse Baltic tribes (mostly Old Prussians, Galindians, and Yotvingians), who slowly moved eastwards mainly under the pressure of the Teutonic State and were finally conquered^27–30^. To trace the changes in vegetation cover and the process of forest exploitation in time and space, connected with human settlement, across the Polish Lowlands during the last 1500 years, it is now opportune to complement the existing knowledge with novel high-resolution palaeoecological studies that have recently become available.

Ralska-Jasiewiczowa^31^ pointed out that the presence and retreat of hornbeam forests were linked to intensive farming. Later, it was noticed that the local history of human settlement and the associated economy were the main reasons for the persistence of hornbeam across northern and central Poland^24^. Although previous studies have indicated the issue of hornbeam expansion during the MP, these did not focus on the precise duration of the process and the subsequent decline of certain species^24,32^. Noryśkiewicz^32^ reported that hornbeam-dominated forests in Chełmno Land (central-northern Poland) developed between the phases of increased human activity, namely between the Roman and Early Medieval periods. According to Makohonienko^23^, the hornbeam can be regarded as a species that accurately represents the scale of human-made clearings and transformation of Polish forests during the last 2500 years.

As pointed out above, hornbeam, as reflected in pollen diagrams, can be a most helpful indicator for tracking forest transformation, especially during the Early Medieval period due to its sensitivity to human impact. Since the influential work by Ralska-Jasiewiczowa et al.^33^, who described plant migration patterns in Poland based on isopollen maps, many records from Polish Lowlands supported by reliable chronologies, have been published. Isopollen maps, despite including many sites were designed in 500-years intervals, which is an insufficient resolution to precisely estimate the timing of regeneration and decline of the natural (or quasi-natural) forests during the Early Medieval and later times. Although several publications which describe the anthropogenic transformation of forests is available^5,8,33^, a concise regional summary of the spatial distribution pattern of the Early Medieval forest decline in the Polish Lowlands is lacking^20^.

To fill this gap, we summarized 36 pollen profiles with reliable chronologies based on radiocarbon dating and spanning the period of regeneration of oak-hornbeam (and beech) forest during the MP and its further retreat. We hypothesize that the diverse development of past societies after the MP on the Polish Lowlands was the main driver of asynchronous forest loss. Such deforestations are distinctly recorded in the pollen records of oak (*Quercus*), beech (*Fagus sylvatica*) and especially hornbeam (*Carpinus betulus*) in the central and north-western Poland as well as spruce (*Picea abies*) in north-eastern Poland^22,24,34–38^. As clearly as the available data permit, we aim to delineate the spatio-temporal trends of Early Medieval forest regeneration and subsequent decline on the Polish Lowlands where the availability of many records enables these trends to be investigated.

## Study area

**Figure 1:**
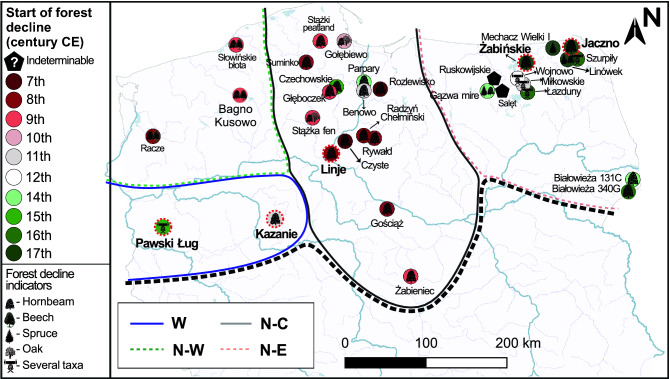
Geographical distribution of sites from the Polish Lowlands representing the beginning of forest decline after ca. 500 cal. CE and indicators of decline. The relevant publications are listed in Table 1. High-resolution data from the sites marked with red dashed circles are presented in Fig. 4. Abbreviations for simplified names of geographic regions used in the text: W—western, N–W—north-western, N–C—north-central, N–E—north-eastern. A black broken line indicates the southern limit of the study area. Map constructed by SC with QGIS 3.16.0 'Hannover' (https://qgis.org/en/site/index.html) and Corel Draw × 8 (https://www.coreldraw.com/en/).

The studied area, i.e. the Polish Lowlands, refers to the Polish part of the central European plain, which extends from central Poland to the Baltic coast (Fig. 1). Most of the area was glaciated during the last glaciation (late Weichselian), ca. 24,000 years ago^39^. However, three sites, i.e., Białowieża 131C and 340G, and Żabieniec are located shortly to the south of the Last Glacial Maximum (LGM) limits. The Polish Lowlands include many lakes and wetlands, making possible the construction of high-resolution palynological records in this part of Poland.

The climate is transitional between oceanic and continental influences^40^. A continental climate characterizes the north-eastern part. Accordingly, it has a greater abundance of coniferous tree species, including *Pinus sylvestris* and *Picea abies*, while the north-western part is characterized by oceanic air masses favouring *Fagus sylvati*ca^41,42^. The western and north-central areas are dominated mainly by oak-hornbeam forests^43^. The present vegetation cover is a result of previous transformations; forestry practices played a significant role and promoted the planting of *P. sylvestris*, which is now the most abundant species across the Polish Lowlands^44,45^.

## Results and discussion

### Spatial and temporal distribution of large-scale forest decline across the Polish Lowlands after the Migration Period

**Figure 2:**
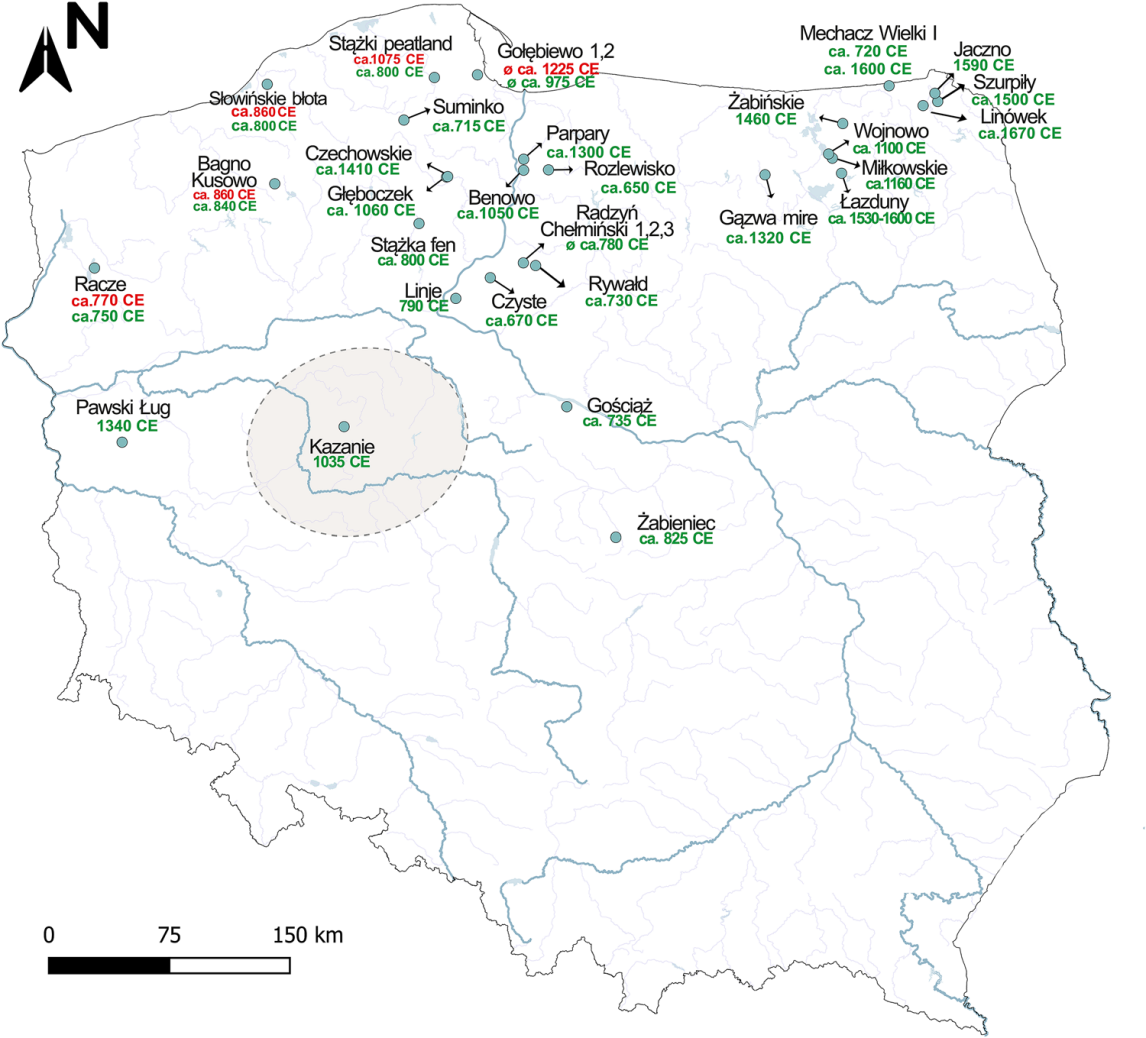
Geographical distribution of sites from the date at which *Carpinus betulus* (green) and *Fagus sylvatica* (red) peaked before decline commenced during the last 1500 years. The area enclosed by a dashed circles indicates the Greater Poland region. Map constructed by SC with QGIS 3.16.0 'Hannover' (https://qgis.org/en/site/index.html) and Corel Draw × 8 (https://www.coreldraw.com/en/).

**Figure 3:**
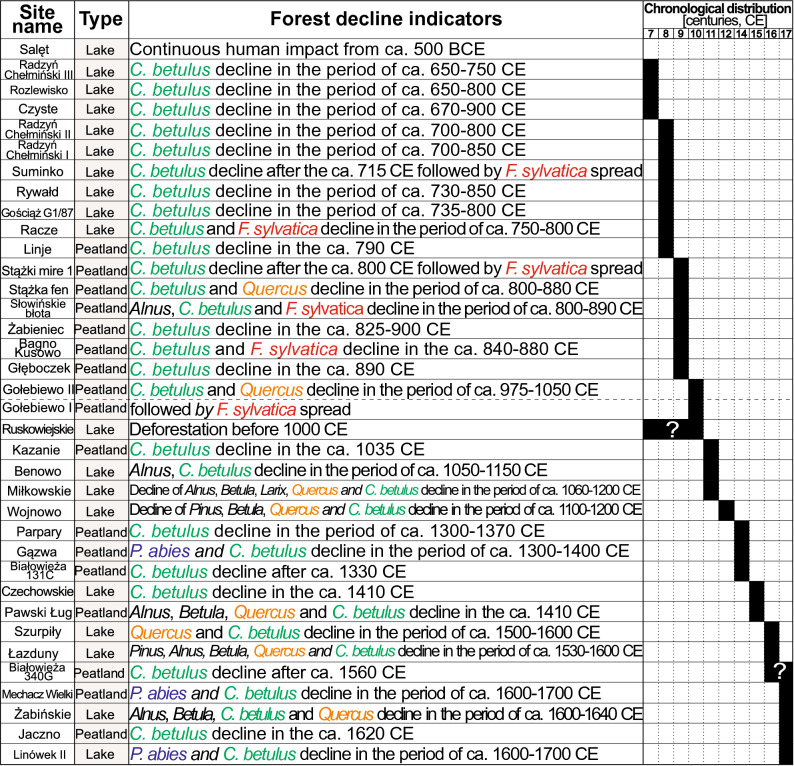
Beginning of forest decline after the Migration Period as recorded at selected sites in the Polish Lowlands during the last 1500 years. Centuries are indicated as i.e. 801–900 CE. For details regarding the sites see Table 1.

The overwhelming majority of the sites across the Polish Lowlands (30 out of 32 studied sites, 34 out of 36 pollen profiles) experienced forest decline after the MP (ca. 360–510). A decline of *C. betulus* percentages was recorded in 34 of the 36 analyzed profiles, while *F. sylvatica* declined at five sites and *P. abies* at three sites (Figs. 2 and 3). On the other hand, the decline of *Quercus* was recorded at eighth sites, which was also accompanied by the decline in other tree species (Fig. 3).

The forest decline was time-consistent in north-western Poland (western Pomerania), where the process took place in the eight to ninth centuries (Figs. 1 and 2)^20,22,46^.

In contrast, western Poland revealed asynchronous decline ranging from the eleventh and fourteenth centuries^47,48^. However, only a few high-resolution studies covering the last 1500 years are available in this region (Fig. 1).

Forest loss started in the seventh century in north-central Poland (Figs. 1, 2 and 3). In this region, in the 16 sites hornbeam was the tree taxon representing the first phase of deforestation (Figs. 1 and 3). Pollen data from the Żabieniec peatland and Lake Gościąż show that, in central Poland, anthropogenic deforestation began as early as the eighth-ninth century^24,49,50^. Data from other parts of this region further to the north, where several fine-resolution profiles are available, show a consistent palynological signature of forest decline, recorded mainly from the seventh to ninth centuries (Figs. 1, 2, and 3). Late local settlement intensification was probably the reason why the two of the sites from north-central Poland recorded a decline during the 13th–14th centuries and do not follow the regional pattern^34,37^ (Figs. 1, 2 and 3). As documented by the data from the Polish Lowlands, the beech optimum occurs mainly after the human-induced hornbeam deforestation^36,46,51,52^, which appears to be related to the selective logging of hornbeam and oak and which facilitated the expansion of beech. During these times in Poland *Quercus* was widely favored for various constructions^53–55^, whereas *C. betulus* was used mainly as firewood^53^.

In north-eastern Poland, two clusters of sites show diverse temporal patterns of forest eradication. *P. abies* showed signs of forest decline at three sites (Figs. 1 and 3). The sites at the north-eastern border of Poland recorded a surprisingly consistent decline pattern during the 16th–17th centuries^38,55–59^. In contrast the remaining sites document pollen-inferred forest decline during (at least) the 10th–16th centuries (Figs. 1 and 3). For the Ruskowijskie site, the timing of the decline of the various tree species is unknown as there are no pollen data available older than ca. 1000^60^. Only Lake Salęt provides a record of continuous impact by human activity, dating from ca. 2500 years and extending to at least the end of the eight century, which was probably related to favorable local settlement conditions^60,61^. Human transformations and clearance of local woodlands were registered from the 11th/12th century in lakes Miłkowskie and Wojnowo located in one of the settlement microregions. These changes can be related to the activities of the Prussian Galinditae people^35^. The sites in Białowieża Forest recorded a forest decline during the 14th–16th centuries (Figs. 1 and 3).

### Diverse spatiotemporal features of human-induced old-growth forest decline deduced from pollen profiles

The summary of pollen records reveals that the north-central and north-western parts of Poland in particular, experienced the widespread expansion of forests dominated by hornbeam and sometimes beech between 600 and 800, i.e. at least a hundred years after the end of the MP (ca. 360–510) in Poland (Supplementary Information, Fig. S1). This expansion ends mainly during the interval 800–1000 (Fig. 2). The forests at the time of human interference soon after the MP could be considered at least as old-growth forests^73^. Kołaczek et al.^76^ showed that the hornbeam optimum was reached ca. 300 years after the decrease of agricultural activity, which points to low a level or indeed lack of human activity that facilitated the expansion of the natural forest. In these regions, the regeneration phase lasted up to several hundred years. This also implies that the scale of human abandonment was of long duration.

The subsequent decline in most of the Polish Lowlands corresponds with the dawn of new societies that gave rise to an early Slavic culture^25^. The few available historical documents indicate that Slavic settlement on Polish lands began as early as the fifth-sixth centuries^77^. This impact has likely gone unrecorded by the high-resolution palynological data due to the dispersed nature of settlement or absence of settlement. According to archaeological data, the settlement of the early Slavic tribal structures expanded several hundred years later, probably in the eighth-ninth centuries^75^. From the eighth to early tenth centuries, Early Medieval strongholds were rapidly established in the western region of the Slavic domain^25,76,77^. This is in line with both the time of the hornbeam and beech expansion after the MP and its abrupt decline during the eighth-tenth centuries (Figs. 1, 2 and 3). Since the beginning of the Medieval Period in Poland (early Slavic phase), there had been a gradual increase in human activity in north-central and north-western Poland. The decline of hornbeam mostly coincided with the rise in agricultural and pastoral activity^22,24,34,37,51,64^.

Due to its high calorific value, low utility value for tool production or construction^53^, and occurrence on fertile habitats preferred for cultivation^78,79^, hornbeam was probably the main tree cut by past societies inhabiting the Polish Lowlands (Figs. 1, 2 and 3). The sites that had the highest abundance of hornbeam experienced its fastest decline (mostly during the seventh–eighth centuries), e.g.: Gościąż, Rywałd, Radzyń Chełmiński, Benowo or Rozlewisko. (Figs. 2 and 3). Moreover, the hornbeam forest decline in north-central Poland was not accompanied by a corresponding decline in other main forest-forming taxa (Fig. 3). This supports the idea that the hornbeam-dominated forests that now occupied the most fertile soils were deforested first because they provided readily accessible material for heating and were highly suitable for farming^34,67^.

**Figure 4:**
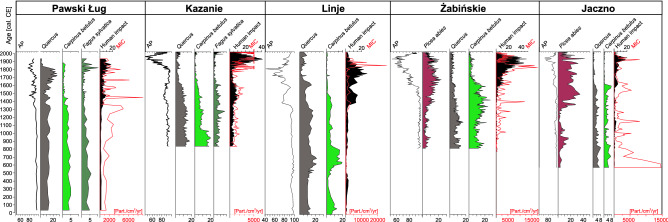
Simplified percentage pollen diagrams from five sites (with secure dating and high-resolution pollen data) located along north-western to north-eastern direction. Selected pollen taxa, composite pollen curves (AP and anthropogenic pollen indicators) and micro-charcoal influx (MIC) are shown.

Timber was the primary material used in the construction of Early Medieval strongholds in Poland^77^. Due to its mechanical properties, oak was mainly used for this purpose^56,57^. In this context, it is worth noting that the abrupt decline of *Quercus* before the eleventh century is recorded in only three sites (Fig. 3). This may signify that, in contrast to hornbeam, oak was conserved as a valuable source of timber. This is supported by pollen data collected from Pawski Ług, Kazanie, and Linje (Fig. 4). In these sites, a rapid decrease of the *Quercus* curve occurs after the sixteenth century, which is several hundred years later than the decrease in the *C. betulus* pollen curve. Livestock grazing, especially pigs in oak and beech woodlands, was common in Poland during the Middle Ages up to early 19th century^44,80^.

In contrast to *C. betulus*, *Quercus* pollen representation remains steady until the end of early modern times (18th century). It may imply that these woodlands (mainly oak; possibly woodland pastures) continued to function despite the increasing economic activity, manifested by the increases of human impact curves in these sites, especially from the 13th–14th centuries onwards (Fig. 4). Pollen curves for *Quercus* (in these three sites) and *F. sylvatica* (in Kazanie mire) do not appear to respond to livestock grazing in woodland pastures, which was widely practiced during the Medieval times throughout Europe^81–83^, including Poland^84^ because woodland pasture did not impinge on overall pollen production by these trees.

Based on a comparison of modern pollen spectra with fossil pollen data, Tobolski^87^ concluded that the old-growth hornbeam forests had no analogues in the present-day oak-hornbeam forests (*Querco-Carpinetum*). In Greater Poland, very high values of hornbeam pollen (including the last optimum phase attributed to MP) were recorded in several sites^87–90^, reaching even as much as 50%^89^. This indicates that the canopy layer of the regenerated forests was dominated by hornbeam. Modern pollen data collected from a small area (32.3 ha) in Greater Poland, which is dominated by oak-hornbeam forest give *Carpinus* values of only 11%^85^. Similar results were obtained from trapping sites (a 12-year pollen monitoring program, Roztocze, south-eastern Poland) located 1–25 m from hornbeam trees. The average percentage values in the two sites were recorded at 11.7% and 2.8%, respectively^90^. Tobolski^85^ also compiled the percentage maxima of *C. betulus* in north-western Poland during the Holocene and selected two clusters of sites in central Greater Poland and Pomerania, respectively, characterized by the highest values. However, the chronology in these sites was based mainly on the palynology; e.g. peaks and declines of hornbeam. According to historical and archaeological data, the first structures of the Polish state were established in Greater Poland. However, except for the Kazanie site (which records the last ca. 1200 years)^48^, high-resolution palynological data covering the last 1500 years are lacking in this region (Fig. 3). Such data are expected to reveal new insights into the environmental history of the beginnings of the Polish State, and so are highly desirable.

The data discussed above suggest that the key indicator/proxy of early Slavic expansion and economic growth is the phase of rapid deforestation (which mainly affected hornbeam) in Poland's north-western and north-central areas that occurred between 800–1000. The loss of extensive natural forest areas during the Slavic expansion can be considered the beginning of the formation of anthroecosystems^9^, and involved processes that significantly accelerated with further development of state structures^47,48^. This pattern, however, was delayed in the north-eastern area of modern-day Poland (formerly Prussia), most likely due to the presence of different types of settlements in the area, which points to a much later forest decline caused by Prussian tribes and, subsequently, by the Teutonic Order^29,91–93^. Due to the generally weaker human influence in this region, the hornbeam optimum was also much later than in the area settled by the Slavs.

In the Polish Lowlands the disappearance of natural forests can also be attributed to influences other than the Slavic migration and expansion. One such area is the north-eastern region, in the Baltic settlement zone. Before the impact of the Teutonic Order, this territory was occupied by several tribes^60^. Though many palynological records exist in these former Prussian lands, radiocarbon-dated profiles were only available from 12 sites until recently^35^. Rapid progress in recent years has resulted in several fine-resolution pollen data sets based on robust chronology from this region (Fig. 2, Table 1). These studies shed light on the various deforestation processes during the last 1500 years. Some areas of the north-eastern region had already been cleared before the influence of the Teutonic Order. Substantial forest decline is recorded in lakes Ruskowijskie and Salęt before the tenth century, as well as in lakes Miłkowskie and Wojnowo before the 11th/12th century (Figs. 1 and 3). These sites reflect the activity of the Prussian Galinditae tribe in two settlement micro-regions, as indicated by archaeological data^27,60^. The subsequent, large-scale forest clearings in the Great Masurian Lake District, which were attributed to the economic activity and intensive colonization of the Prussian territory by the Teutonic Order, is recorded as late as the 17th century in Lake Łazduny and slightly later (since 1610) in Lake Żabińskie (Figs. 1 and 3). According to pollen data, the north-eastern edge of the former Prussian land (Suwałki Lake District) was the last to escape the effects of what was to become sustained economic expansion (Figs. 1 and 3). This region, which was primarily colonized by the Jotvingan Baltic tribe, was largely depopulated in the 13th century, due to the military campaign/crusade of the Teutonic Knights^91–93^. Despite pollen data suggesting a minor impact of the Baltic tribes on the environment since the ninth century onward^56,58^, the forest composition seems to be modulated by climatic factors, which is indirectly confirmed by the microcharcoal curves in the north-eastern edge of Poland (Fig. 4) as well as the simultaneous low representation of anthropogenic taxa. On the other hand, fires could have been caused by human activity, for example, to facilitate capturing animals. Microcharcoal data from Jaczno and Mechacz Wielki bogs, as well as Lake Żabińskie^56,72,94,95^, suggest that during the Medieval Warm period (800–1300) higher fire frequency was probably one of the causes of the retreat of *P. abies* at the limit of its Polish Holocene distribution range^96^. The most severe changes in this region were recorded as late as the 16th and 17th centuries and are reflected mainly in the decline of *C. betulus* and *P. abies*. The demise of these forests took place later because, for various reasons, they escaped excessive timber harvesting for long periods^58,91^.

**Table 1:** List of sites from the Polish Lowlands with pollen profiles used in this study. Sites are listed according to the chronology of forest decline.

Site name	Year of first publication	Resolution (years)^a^	Altitude (m a.s.l.)	Length of record (cm)		Radiocarbon dates (no. and type)	Other dating
				Complete profile	Last 1.5 ka years		
Salęt^60,61^	2014	ca. 24	129	430	ca. 195	6 (0, 2, 2), AMS	^210^Pb
Radzyń Chełmiński III^34^	2019	ca. 21	79	ca. 100	ca. 100	12 (1, 6, 5), bulk	
Rozlewisko^34^	2019	ca. 26	42	ca. 57	ca. 57	6 (0, 4, 2), bulk	
Czyste^20,32^	2013	ca. 40	73	ca. 260	ca. 140	6 (0, 1, 2), bulk	
Radzyń Chełmiński II^34^	2019	ca. 27	79	ca. 80	ca. 55	8 (0, 5, 1), bulk	
Radzyń Chełmiński I^34,62^	2019	ca. 20	79	104	104	11 (0, 7,4), bulk	
Suminko^36^	2015	ca. 39	163	1050	ca. 230	13 (0,1,1), AMS	^137^Cs
Rywałd^34^	2019	ca. 28	90	ca. 110	ca. 95	10 (1, 6, 2), bulk	
Gościąż G1/87^24,49,63^	1998	ca. 55	64	1700	ca. 470	16 (0, 0, 0), bulk	Varvochronology
Racze^20^	2020	ca. 19	23	144	144	8 (0,3,1), AMS	
Linje^64^	2015	19	91	210	172	20 (9, 4, 5), AMS	^210^Pb
Stążki mire 1^52^	2011	ca. 55	215	110	110	5 (2, 2, 1), AMS	
Stążka fen^65,66^	2012	ca. 14	100	110	110	8 (4, 1, 3), AMS	
Słowińskie błota^46^	2009	12	29	100	100	10 (3, 2, 5), AMS	
Żabieniec^50^	2009	ca. 62	180	180	ca. 120	3 (0, 2, 1), AMS	
Bagno Kusowo^22^	2015	ca. 50	145	800	290	9 (0, 2, 1), AMS	
Głęboczek^67^	2019	100	137	400	ca. 110	44 (3, 2, 4), AMS	^210^Pb
Gołębiewo II^51^	2016	ca. 62	125	235	ca. 127	7 (2, 1, 0), AMS	
Gołębiewo I^36^	2016	ca. 29	125	355	ca. 120	12 (1, 2, 0), AMS	
Ruskowijskie^60^	2016	ca. 27	142	40	40	3 (0, 1, 1), AMS	
Kazanie^48^	2021	7	100	171	171	18 (13, 4,1), AMS	^210^Pb
Benowo^34^	2019	ca. 20	52	ca. 90	ca. 90	14 (7, 4, 3), bulk	
Miłkowskie^35^	2012	ca. 65	125	ca. 1400	ca. 490	15 (0, 1, 2), AMS	
Wojnowo^35,68^	2012	ca. 40	115	ca. 1050	ca. 260	7 (0,1,1), AMS	
Parpary^34^	2019	ca. 20	58	ca. 50	ca. 50	12 (3, 7, 1), bulk	
Gązwa^69^	2017	ca. 32	155	900	ca. 245	9 (1,1,1), AMS	
Białowieża 131C^70^	2015	ca. 26	164	ca. 73	ca. 72	5 (1, 1, 2), AMS	^210^Pb
Czechowskie^37,71^	2019	5	108	No data		21 (8, 6,4), AMS	Varvochronology, 1875 Askja tephra, ^137^Cs
Pawski Ług^47^	2020	24	122	400	330	39 (19, 7, 3), AMS	
Szurpiły^57^	2019	48	183	ca. 780	ca. 260	14 (2, 2, 1), AMS	Varvochronology, ^210^Pb, ^137^Cs
Łazduny^35^	2012	ca. 56	129	350	ca. 200	7 (2, 1, 0), AMS	
Białowieża 340G^70^	2015	ca. 31	156	ca. 74	ca. 47	4 (2, 0, 1), AMS	^210^Pb
Mechacz Wielki^56^	2017	ca. 18	190	550	ca. 420	9 (2, 2, 3), AMS	
Żabińskie^59,72^	2016	6	117	595	ca. 430	29 (12, 9, 2) AMS	Varvochronology
Jaczno^58^	2020	15	177	396	396	21 (8, 8, 5), AMS	
Linówek^38^	2014	ca. 32	200	700	ca. 240	2 (1,1,0), AMS	

No. of ^14^C dates listed as follows; all dates in profile; dates (in parentheses) relating to intervals (CE timescale): 2–1.5 ka, 1.5–1 ka and 1–0.5 ka; and type of date, i.e. AMS or bulk sediment-derived.

^a^Resolution refers to the average temporal resolution samples from ca. 500 cal. CE.

Although distinct traces of human activity were recorded by pollen data and attested to by archaeological data in the Białowieża Forest, which is considered the last primeval forest in Poland and one of the few such in Europe, human impact does not appear to have caused a break in forest cover continuity; indeed, the forest seems never to have been entirely cut^70,97,98^. Forest clearances occurred in the fourteenth (site BIA/131C) and post-sixteenth centuries (site BIA/340G). However, determination of the spatial extent and duration is challenging due to the small size of the basins that were sampled so that their local pollen source areas are local, and the small number of radiocarbon dates, especially in the interval ca. 500–1500.

### The problem of data aggregation. Specifics of the site versus general patterns

The temporal variations of pollen-inferred forest decline are determined by the degree of landscape transformations at local and also regional levels brought about by human activity and do not always display a uniform signal even among neighboring sites. For example, the sites located close to each other (those within a few square kilometers, such as Lake Salęt, Ruskowijskie, and Gązwa) exhibit completely different palaeoecological signals and hence different times of forest decline (Figs. 1 and 3). A similar phenomenon is also observed at lakes Miłkowskie, Wojnowo and Łazduny (separated by up to several kilometres), where the beginning of permanent deforestation differs by several hundred years (Fig. 1). On the other hand, the local impact recorded in palaeoenvironmental reconstructions at Głęboczek peatland^67^ and Lake Czechowskie^37^ (5 km apart) may explain why two closely located sites in north-central Poland have a different pattern of forest decline. In the Głęboczek site, the decline of hornbeam-marked forest occurred in the early eleventh century and is probably associated with local Slavic society. At the same time, in Lake Czechowskie, which reflects the regional signal, the decline is recorded ca. 400 years later and is related to the economic intensification of the Teutonic Order linked with the development of towns in this area since the fourteenth century^37,71^. The activity of the Teutonic Order is also associated with late forest eradication near the Parpary site^34^, which was recorded as late as the beginning of fourteenth century (Figs. 1 and 3). The late pattern of forest decline observed at the other sites in this region may have resulted from differences in settlement history at the particular sites.

These considerations derived, simplistically, from differences between the sites (e.g. type of site, size, proximity to the settlements, orography and catchment) must be considered when formulating general findings on patterns of old-growth forest loss after the MP and should be brought to bear when interpreting palaeoecological record. Nevertheless, despite intrinsic differences between the sites, forest decline after the MP seems to be influenced mainly by the settlement processes of varying intensities. Taken together, it points to well defined patterns, such as hornbeam-marked deforestation and similar timing of forest decline in particular regions.

## Methods

We selected 36 pollen profiles for our study from the Polish Lowlands. Mostly, we retrieved data from the authors and original publications. Only one of these sites was used in the history of vegetation in Poland based on isopollen maps^33^. In the case of pollen diagrams for which numerical data are not available, the forest decline patterns were reconstructed based on careful visual study of published pollen diagrams (Table 1). Moreover, we also used a few sites from the Neotoma database^65^ (Table 1, Supplementary Information; Table S1). The main criteria for palynological site selection were the availability of at least two^14^C dates for the profile sections representing the last 1500 years or a varve-based chronology. In most of the sites (84.6% of the selected group) a minimum of two dates per millennium for Bayesian age-depth models, was fulfilled as recommended by Blaauw et al.^99^. The chronologies for the selected parts of profiles followed original works. We chose sites where the temporal resolution of sampling is no higher than 70 years. However, we included the Głęboczek site (temporal resolution 100 years) because of its reliable chronology. In the case of Greater Poland, however, we include sites that do not fulfil the chronological criteria indicated above, because in this region the highest *Carpinus* pollen representation in the Polish Lowlands has been recorded^87^ (Fig. 2, Supplementary Information; Fig. S1 and Table S1). Most of the sites used in this synthesis have records that extend back to before the MP but there are eight sites with records that do not go back more than 1500 years. Ages are cited in this paper as a calibrated year or century CE. Furthermore, we show a general pattern of forest expansion and its subsequent decline based on the location of sites along the west–east gradient.

Because of the different interpretations of percentage threshold values of regional importance^100^, which is aim-dependent and/or related to site-specific features, we focused on the last pollen percentage maximum value (during the past 1500 years) of hornbeam and beech optimum to approximate the start of the forest decline (Supplementary Information; Fig. S2). We took into account sites where pollen percentage maxima exceeded 5%, i.e. values that suggesting the local presence of both taxa^33,101^. The declines of the taxa mentioned above were considered only when a decrease of arboreal pollen (AP) was observed, so that bias arising solely from the compositional change of tree taxa is avoided. To provide a more comprehensive account of forest decline we have supplemented our descriptions with accounts of other arboreal taxa that revealed significant site-specific declines.

## Conclusions

As far as we are aware, this is the first work that summarizes the spatio-temporal variation in post-Migration Period forest decline based on recent, mostly high-resolution palynological data in Central Europe. With reliable age-depth models, these records can be expected to add to our understanding of the specific details of the anthropogenic old-growth forest loss and its spatiotemporal patterns.

We show that human activity favoured the clearance of hornbeam in the first place, which is evidenced by its significant decline in most pollen profiles across the Polish Lowlands. This decline can be considered not only as a local marker for the beginning of a new settlement but also as indicative of an irreversible loss of natural forest. In most of the sites, particularly those in north-western and north-central Poland, the forest decline was probably related to the emergence of new early Slavic culture and Baltic tribes in its northeastern part.

Slavic culture is often described as primitive by academics^75^. Nevertheless, the expeditious deforestation, which occurred mainly within as little as two centuries (800–1000) in north-western and north-central Poland might suggest rapid demographic and economic growth of societies that inhabited this area before establishing the earliest state structures dating to the tenth century.

The most recent human-made degradation of forests was recorded in north-eastern Poland, where the remnants of extensive old-growth forests now serve as poor but essential reminders of their former extent. Because of their uniqueness, resulting, among other things, from their longevity which gives unique insights into long-term ecological processes, we should make every possible effort to preserve them. However, there are no analogues of the pristine hornbeam-dominated forest in Poland, which underwent significant change as a result of widespread and severe deforestation in the Medieval period.

These massive deforestations in north-eastern Poland occurred in most sites no earlier than the fourteenth century. This implies that the time of forest clearance in individual sites varied by up to 900 years. This, in turn, is related to the settlement processes of varying intensities in different parts of the Polish Lowlands.

This work offers a promising application of palynological data, especially as it complements the limited archaeological knowledge of the early Medieval Period in the Polish Lowlands. For example, these data can be used to estimate the interval between the first evidence of newcomers, and the development and expansion of economic activities that led to abrupt and often major deforestations.

## Supplementary Information

Supplementary Information.
