# Supplementary material for: Synthesis of palaeoecological data from the Polish Lowlands suggests heterogeneous patterns of old-growth forest loss after the Migration Period
Source: Sci Rep. 2022 May 20;12:8559. doi: 10.1038/s41598-022-12241-1 (PMC9122992; doi:10.1038/s41598-022-12241-1)
Supplement: Supplementary file 1 — Supplementary Information. [file 41598_2022_12241_MOESM1_ESM.docx]

Supplementary Information

**Synthesis of palaeoecological data from the Polish Lowlands suggests heterogeneous patterns of old-growth forest loss after the Migration Period**Czerwiński et al.


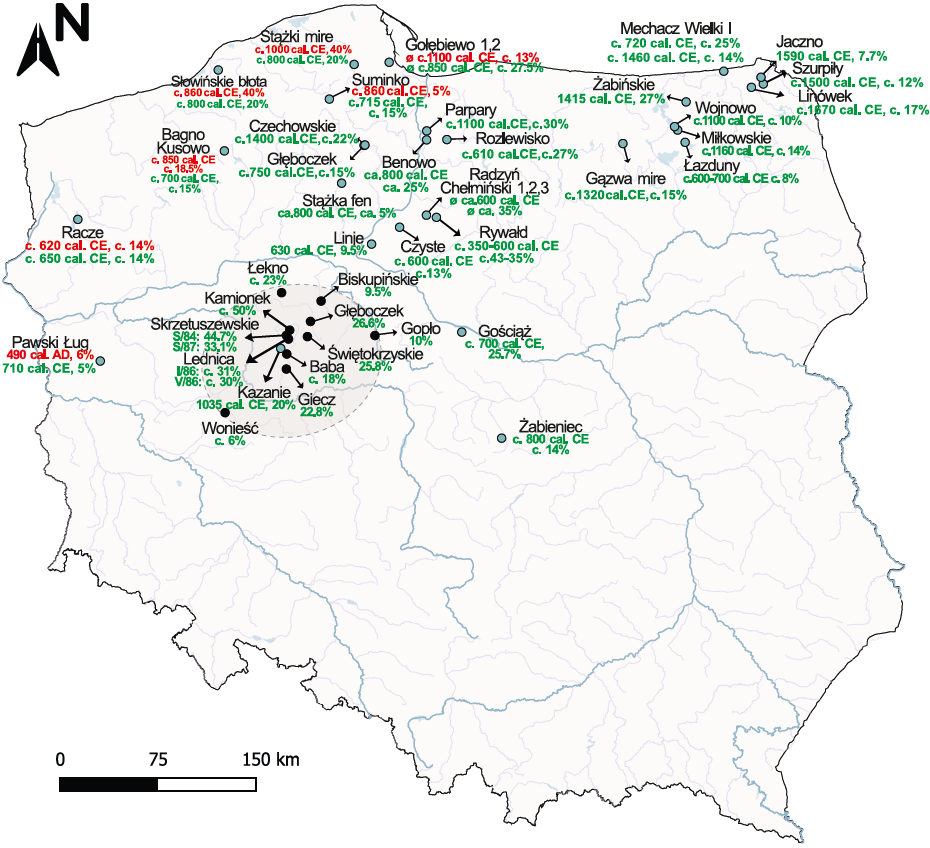


**Fig. S1** Geographical distribution of sites from the Polish Lowlands showing the highest peak of *Carpinus betulus* (green) and *Fagus sylvatica* (red) pollen during the last 1500 years. The dashed circle indicates the sites from the Greater Poland region listed in Table S2. Map constructed by SC with QGIS 3.16.0 'Hannover' (<https://qgis.org/en/site/index.html>) and Corel Draw x8 (https://www.coreldraw.com/en/)

**Table S1** References to sites from the Greater Poland region showing the highest peak of Carpinus betulus pollen during the last 1500 years

| **Site name** | **Citation** |
| --- | --- |
| Kamionek | Filbrandt ^1^ |
| Lednica I/86 | Makohonienko ^2^, Neotoma database |
| Lednica V/86 | Litt and Tobolski ^3^, Neotoma database |
| Głęboczek | Makohonienko ^4^, Neotoma database |
| Świętokrzyskie | Makohonienko ^4^, Neotoma database |
| Biskupińskie Lake | Noryśkiewicz ^5^, Neotoma database |
| Skrzetuszewskie S/84 | Tobolski ^6^, Neotoma database |
| Skrzetuszewskie S/87 | Tobolski ^7^, Neotoma database |
| Baba | Milecka ^8^, Neotoma database |
| Giecz 2/90 | Milecka ^9^, Neotoma database |
| Łekno | Milecka ^10^ |
| Wonieść | Dörfler, et al. ^11^ |

References:

1 Filbrandt, A. in *Wstęp do paleoekologii Lednickiego Parku Krajobrazowego* (ed K. Tobolski) (Wydawnictwo Naukowe Uniwersytetu im. Adama Mickiewicza w Poznaniu, 1991).

2 Makohonienko, M. in *Wstęp do paleoekologii Lednickiego Parku Krajobrazowego* (ed K. Tobolski) (Wydawnictwo Naukowe Uniwersytetu im. Adama Mickiewicza w Poznaniu, 1991).

3 Litt, T. & Tobolski, K. in *Wstęp do paleoekologii Lednickiego Parku Krajobrazowego* (ed K. Tobolski) (1991).

4 Makohonienko, M. *Przyrodnicza historia Gniezna*. (Homini, 2000).

5 Noryśkiewicz, B. in *Zarys zmian środowiska geograficznego okolic Biskupina pod wpływem czynników naturalnych i antropogenicznych w późnym glacjale i holocenie* (ed W. Niewiarowski) 147-179 (1995).

6 Tobolski, K. in *Wstęp do paleoekologii Lednickiego Parku Krajobrazowego* (ed K. Tobolski) (Wydawnictwo Naukowe Uniwersytetu im. Adama Mickiewicza w Poznaniu, 1991).

7 Tobolski, K. Paläoökologische Untersuchungen des Siedlungsgebietes im Lednica Landschaftspark (Nordwestpolen). *Offa* **47**, 109–131 (1990).

8 Milecka, K. *Historia działalności człowieka w okolicach Giecza i Wagowa w świetle analizy pyłkowej*. 43-95 (1998).

9 Milecka, K. in *Wstęp do paleoekologii Lednickiego Parku Krajobrazowego* (ed K. Tobolski) (Wydawnictwo Naukowe Uniwersytetu im. Adama Mickiewicza w Poznaniu, 1991).

10 Milecka, K. in *Mechanisms of anthropogenic changes of the plant cover* (eds B. Jackowiak & W. Żurkowski) (Bogucki - Wydaw. Naukowe, 2000).

11 Dörfler, W., Hildebrandt-Radke, I., Spychalski, W. & Lutyńska, M. *Zapis palinologiczny, litologiczny, geochemiczny i diatomologiczny regionalnych zmian użytkowania terenu w osadach jeziora Wonieść (Pojezierze Wielkopolskie)*. Vol. Tom V (Wydawnictwo Nukowe UAM, 2009).

**
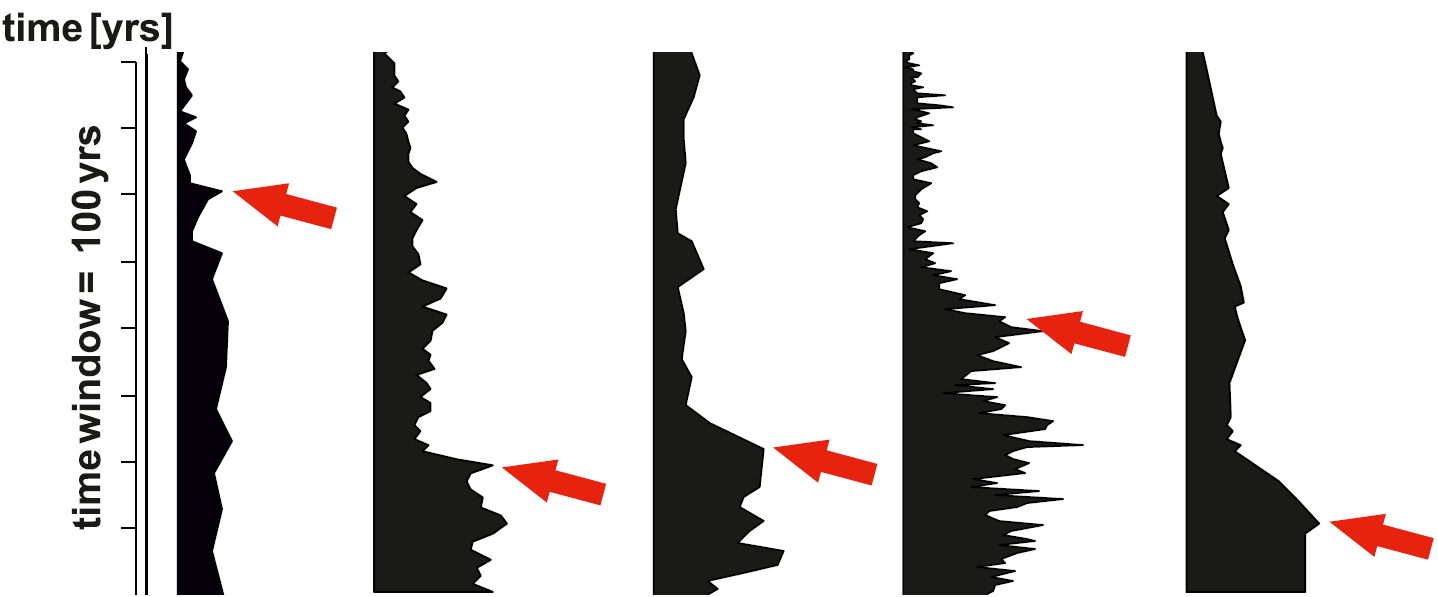
**

**Fig. S2** Schematic examples of the most recent pollen percentage maximum value (red arrow), which were interpreted as forest decline. Minimum value before decline is 5%.

**Table S2.** List of taxa used for anthropogenic pollen indicators in Figure 4

| *Agrostemma githago*, *Ambrosia* t., *Artemisia*, *Avena* t., Brassicaceae undiff., Chenopodiaceae, *Centaurea cyanus*, *Cerealia* undiff., *Convolvulus arvensis*, *Fagopyrum*, *Hordeum* t., *Linum usitatissimum*, *Phacelia tanacetifolia*, *Plantago major*/*major*, *Polygonum aviculare* t., *Rumex acetosa* t., *Rumex acetosella* t., *Rumex cf. obtusifolius*, *Plantago lanceolata*, *Triticum* t., *Secale cereale*, *Scleranthus annuus*, *Scleranthus perennis* |
| --- |

t. = type
